# Women migrant workers’ (WMWS) deskilling

**DOI:** 10.12688/openreseurope.18205.1

**Published:** 2024-08-21

**Authors:** Marie Ruiz, Stellamarina Donato

**Affiliations:** 1 Universite de Picardie Jules Verne, Amiens, Hauts-de-France, France; 2 Libera Universita Maria Santissima Assunta, Rome, Lazio, Italy

**Keywords:** migration, gender, education, skills

## Abstract

Women are key actresses in sending and receiving countries’ developments, and their contributions includes social and financial remittances, education and the transmission of social and cultural values (United Nations, 2006). Despite, women migrants’ undeniable contributions, they too often undergo deskilling, a process defined as the employment of workers in a different field or below their qualifications. The factors influencing deskilling include the lack of recognition of skills and qualifications, difficult access to information and employment opportunities, lack of support in the destination country and linguistic barriers.

Migration can impact the social mobility of women migrants, yet not always positively (Nowicka, 2012). In the labour market, women migrants are generally disadvantaged because of occupational gender segregation, the lack of network support and childcare responsibilities (EU Commission, 2022), with higher risks of deskilling and downward social mobility. The objective of the brief is to shed light on WMWs’ deskilling and explore the impact of gender and ethnicity in labour segmentation, de-emancipation being a consequence of WMWs’ deskilling and overrepresentation in reproductive unskilled jobs.

## Introduction

2023 was marked by the European Year of Skills meant to help “people get the right skills for quality jobs and supports” ( European Year of Skills, 2024) by encouraging upskilling in order to boost sustainable growth with a competitive workforce and meet the 2030 social target of 60% EU people in training and 78% employed. Yet, this initiative to boost skills’ development is challenged by underlying deskilling, which affects women migrant workers (WMWs) in greater numbers than men. Deskilling concerns the devaluation of workers’ qualifications employed in occupations below their skills, training, expertise and professional experience, and it is highly relevant to gender and migration studies. Indeed, a 2020 Eurostat survey concludes that WMWs are at a higher risk of being overqualified, EU WMWs being deskilled at 6% more than men, and 6.4% for non-EU born WMWs. Quantitative data confirm that in 2019, 1/5 highly educated WMWs experienced deskilling: 40.7% for migrant women compared to 21.1% for national women in 2020. Deskilling rates are higher in Southern Member States as Greece, Italy, Spain and Cyprus, overqualified WMWs being most represented in Italy (47.8%), Cyprus (47.7%) and Spain (47.2%) ( Eurostat, 2024).

The objective of this brief is to shed light on WMWs’ deskilling and explore the impact of gender and ethnicity in labour segmentation, de-emancipation being a consequence of WMWs’ overrepresentation in unskilled reproductive jobs. WMWs in sectors such as health, care, and education are particularly affected by deskilling.

## General framework

Deskilling is a work-related issue that is impacted by an international and EU framework of policies on labour. Among the main policies and decisions that are meant to provide protection to women migrant workers, at **international level:**

- The 1979 **Convention on the Elimination of all Forms of Discrimination Against Women (CEDAW)** contains recommendations for the protection of WMWs, especially recommendation No. 26 (2008) aiming at protecting WMWs’ rights;
- The 2011 **ILO Domestic Workers Convention (No. 189)** guarantees decent work conditions and labour rights to domestic workers and, due to their above-mentioned overrepresentation in this field, it is key to WMWs, especially General Comments No. 1 (2011) on Migrant Domestic Workers and No. 2 (2013) on the rights of migrant workers in an irregular situation. Previous ILO Conventions include: Migration for Employment Convention (Revised), 1949 (No. 97); Equal Remuneration Convention, 1951 (No. 100); Discrimination (Employment and Occupation) Convention, 1958 (No. 111); Migrant Workers (Supplementary Provisions) Convention, 1975 (No. 143); and accompanying recommendations, Private Employment Agencies Convention, 1997 (No. 181);
- The 1990 UN **International Convention on the Protection of the Rights of All Migrant Workers and their Families (ICRMW)** was implemented in 2003 to protect migrants’ rights, including women and families, and prevent discrimination against migrants in the workplace;
- The 2016 **New York Declaration for Refugees and Migrants** seeks to address women migrants’ vulnerabilities and underlines the need to acknowledge women migrants’ skills and promote their empowerment;
- The **2030 Agenda for Sustainable Development** provides for protection for WMWs, with such goals as Goal 4 on education, Goal 5 on gender equality, Goal 8 on decent work, Goal 10 on reducing inequality, and Goal 17 on global partnerships. For instance, goal 5.4 recognizes “the value of unpaid and domestic work through the provision of public services and social protection”, which is key for WMWs in the care industry, and goal 8.8 underlines their necessary protection in precarious employment.

At **European level**, in 2011 the Council of Europe adopted

- **Recommendation CM/Rec(2011)2** of the Committee of Ministers to member states on validating migrants’ skills and acknowledged “that many migrants’ skills, competences and qualifications, howsoever acquired, are still not properly validated or recognised, and that as a result they cannot gain employment corresponding to their skills, competences and qualifications, and [was] concerned about the consequent waste of their human capital to society and to themselves” ( Council of Europe, 2024), hence aiming at curbing deskilling in the interest of European Member States and individual migrant workers;
- Introduced in 2017, the **European Qualifications Passport for Refugees** (EQPR) was based on the Lisbon Recognition Convention and meant to identify refugees’ skills to curb deskilling by easing access to higher education. Today, 22 countries are involved in the project supported by the UNHCR.

At **EU level**, the following directives and policies apply to WMWs:

- The Council of Europe’s above-mentioned EQPR has been complemented by a joint EU project titled “ **Supporting an efficient national mechanism of recognition of refugees’ qualifications**”, to assist Italy where the EQPR is most applied;
- Part of the European Pillar of Social Rights (20 key principles for social cohesion and inclusiveness introduced in 2017 at the Gothenburg Summit), the **Work-life Balance Initiative** can ease WMWs’ work conditions by providing for childcare, parental leave, by combatting discrimination in the workplace, and by developing incentives for mothers to get decent employment, and fight against the “motherhood penalty” ( Riaño, 2021), i.e. household and childcare family duties which affect WMWs’ access to employment without a network to support them in the receiving nation.
- The 2023 **European Year of Skills** was meant to develop workers’ skills and thus curb deskilling, yet it is too early to assess its effects.

Despite the above-mentioned initiatives, there is a need for further policies and directives at EU level to curb deskilling and make more efficient use of WMWs’ skills.

## Deskilling in context and practice

The definition of skills is globally shifting and fluid, depending on the level of education, training, or experience, but above all skills are defined in terms of the receiving nations’ needs, which impacts WMWs’ integration when receiving countries focus on IT and engineering, underrepresented by women, and neglect invisible soft skills, although they are necessary to build socially stable societies ( Purkayastha, 2023). Skills being a fluid social construct based on stereotypes, be they gendered, ethnic or societal, it can take different definitions depending on the period, context and country. The focus on economic needs can have an impact on women who are traditionally discriminated against as pertaining to the reproduction sector (the opposition between productive and reproductive work was introduced after the Industrial Revolution and has entrenched the gender labour divide by confining women to reproductive activities and men to the production realm). A 2017 UN Women report shows that labour migration is highly gendered, based on the gendered productive/reproductive binary, with a higher representation of women in the care industry ( UN Women, 2017).

Deskilling is the underemployment of migrant workers who experience downward social mobility in the receiving nation by taking up jobs with a lower social status and recognition. Over-qualified, their skills can be degraded ( IOM, 2015), which impacts WMWs’ emancipation and well-being. Deskilling is generally the result of administrative barriers to qualification equivalence and accreditation, gender and ethnic stereotypes on WMWs’ skills, and language obstacles, which lead to an erosion of skills and underuse of competences and formal training. According to Kofman, “Deskilling […] often results in the move from one gender order to another” ( Kofman, 2012).

In a briefing published in 2023 by the EU parliament, deskilling is linked to the barriers imposed through exclusionary policies that limit women migrants’ access to employment and emancipation in a receiving nation. The brief reaffirms the European Institute for Gender Equality (EIGE)’s conclusions that women migrants are at a high risk of unemployment and deskilling in the EU ( Orav, 2023). Additionally in 2021, 25% of foreign-born women reported facing difficulties when trying to get a job in the EU ( Orav, 2023). According to Kofman, in 2004 foreign-born women were underrepresented in highly-skilled occupations as compared to nationals except in Ireland, the UK and Hungary. She also concludes that Southern European countries, except for Portugal, tend to present higher levels of deskilling ( Kofman, 2012).

Highly-skilled women migrants are generally present in the care and education sectors, often migrating with their husband, a group referred to as “trailing” migrants. For instance, in the UK the proportion of foreign-born nurses increased by 92% between 1997 and 2004 ( Kofman, 2012). In the EU in 2003–4, WMWs were mostly represented in sales (12.6% of the total employed), hospitality and education (8.1%), health (17%) and households (6.2%) (Kofman, 2009). Between 2003 and 2004, the proportion of overqualified employed foreign-born women (i.e. deskilled) reached up to 53.4% in Greece, and 62% of non-OECD born women. Similarly, Spain reached 47.6% deskilled foreign-born women and 56.7% non-OECD born. These figures stand high as compared to Hungary or Switzerland with respectively 10.5% and 13.8% of foreign-born deskilled women for 8.9% and 19.8% of non-OECD born WMWs ( Kofman, 2012). In 2007, the global proportion of highly-skilled WMWs was EU-born at 23.3%, and non-EU born at 20.7%, with a lower proportion of native-born highly skilled WMWs of 18% (Huber, 2010). Between 1994 and 2004 the disproportion between natives and foreign-born women employed in household services was entrenched, with an increase from 27.1 to 36% for Spain, and 35% to 42.4% for Greece. Northern countries such as the UK and Denmark are marked by a higher proportion of WMWs in the health sector ( Kofman, 2009).

### Factors contributing to deskilling

WMWs are more likely to be deskilled or unemployed than any other group in the EU, and this is because they can be doubly discriminated for their ethnicity and gender. Furthermore, women migrating with families have a lower employment rate: women refugees being 45% likely to be employed as compared to 62% for refugee men ( EIGE, 2020).

Racial and gender stereotypes can also contribute to assigning women to specific roles and jobs as studies show a higher proportion of deskilling or unemployment among WMWs from Africa, Latin America and Asia who are 6 times more likely to be excluded from the labour market, and this underlines the impact of geographical origins in employment in the receiving nations (Gerber, 2019). The labour market has been seen as a “site of class reproduction” ( Bauder, 2006), the native population being given the priority in employment opportunities. Hence, deskilling can act as a domination tool meant to exclude and marginalize non-nationals for instance by not recognizing their qualifications and credentials ( Siar, 2013).

Facing double discrimination, WMWs can be affected by labour gender segmentation. ILO 2013 data indicates that women migrants represent 73% of domestic workers in the world ( EIGE, 2020), often illegally employed, they can be the victims of exploitation and abuse. A 2024 UN Women report shows that WMWs are mostly employed in service and retail (18.8%), elementary occupations (17.3 per cent), craft and related trades (15.2 per cent), professionals (13.9 per cent) and clerks (12.3 per cent) ( UN Women, February 2024).

Hence the factors contributing to WMWs’ deskilling include:

- National schemes for educated migrants favouring IT, engineering and finance graduates, which is a sector less occupied by women ( EIGE, 2020);
- The difficulties to get qualification and educational recognition in the EU particularly affects non-EU migrants, the country of origin being a key determinant, as global South migrants are more likely to face unrecognition of their qualifications.
- Women migrants’ employability can be limited by their childcare responsibility and household duties, their caretaking duties preventing them from accessing language and training courses, a phenomenon referred to as the “motherhood penalty” ( Riaño, 2021). Furthermore, they can be perceived as less competent and available than non-mother migrants, which impacts their employability and remuneration ( Riaño, 2021).
- Lack of knowledge of the job market and limited access to information can prevent WMWs from accessing adequate employment. WMWs do not enjoy the support of family and social networks that nationals do and this is an impediment to their integration in the job market ( IOM, 2015).
- Labour protection deficiencies affect men and women migrants alike and often forces them into precarious and unregulated employment. Scholars have shown how WMWs face “linguistic penalty” in the receiving nations’ job market, discriminated against because of their language skills and “double disadvantage as foreign-born and women ( IOM, 2015).
- Once the WMW has overcome the qualification recognition, she may still face discrimination in the career advancement, a problem which one of WEMov interviewees, D., calls the “migrant glass ceiling” (see below).

### Gender pay gap

If educational and social backgrounds impact WMWs’ integration, UN Women finds that “labour segregation and pay gaps are common among all skill groups” ( EIGE, 2020). In 2020, the global gender pay gap was estimated at 20% ( UN Women, 2020), and a 2023 UN Women report indicates that women earn 24% less than men for the same work, and that women are more represented than men in vulnerable and unprotected employment with 50.5% in 2011 ( UN Women, 2023). In the EU, the gender pay gap was estimated at 13% in 2022, despite an increase in women’s employment reaching 66.2% in 2020 and increased representation in higher education ( EU Commission, 2022).

Riaño bases her 2021 case study of WMWs’ skills on Switzerland because the country’s population counts 25% of foreign-born residents, the 2 ^nd^ largest foreign-born population OECD country, and a high proportion of highly skilled incomers. Interestingly, WMW have a higher level of education than Swiss-born women and come from the EU at 44%, and non-EU countries at 41%. This attractiveness can be explained by low deskilling rates and remarkable advances in gender equal education and employment opportunities, despite the remaining gender pay gap. However, closer investigation shows that WMW have the lowest income of all groups under study, followed by Swiss-born women workers, foreign-born male workers and Swiss-born men workers, which shows the extent of the gender pay gap irrespective the country of origin. Furthermore, women workers face greater deskilling and obstacles in employment, and are even more affected when coming from non-EU countries ( Riaño, 2021).

## WEMov’s approach

### WMWs’ skills and resources

According to the director of the Ödos reception center interviewed by WEMov in Spain in January 2024, WMWs are highly resourceful and they can bring skills that are necessary for the development of the EU. Yet, WEMov considers that WMWs’ skills are underexploited because of deskilling. They acquire soft skills through the migration process and these, beyond their initial training and backgrounds, are key to develop human capital in receiving nations.

Half the 258 million migrants are women, who outnumber men in all regions but Africa and Asia, they thus represent a remarkable workforce ( UN Women, 2024). Despite the gender pay gap and discrimination in the labour market, WMWs were responsible for half the $601 billion remittances in 2016 ( UN Women, 2024). Indeed, WMWs contribute to their sending and receiving nations, notably with social and financial remittances; social remittances concerning the transfer of social capital and values. Although remittances data are not sex-disaggregated, commentators agree that women contribute more than men ( UN Women, 2017).

A 2024 UN Women report states that empowering WMWs is necessary to meet the 2023 Agenda for Sustainable Development, reach gender equality (Goal 5), decent work conditions (Goal 8), end poverty (Goal 1), ensure food security (Goal 2) and health (Goal 3), and reduce inequalities (Goal 10) ( UN Women, 2024). The report also shows that easing women’s access to employment can boost economic development and that gender gaps cost 15% in GDP.

### WEMov’s case studies

A key issue in deskilling is the psychological impact on WMWs, which WEMov has noted in its investigations that exploitation in the workplace and deskilling were not uncommon. For instance, WMWs may be constrained to accept undervalued positions, extra work hours and lower salaries to secure a work permit and regular position in a welcoming country. Cases of employers abusing WMWs vulnerability in precarious jobs is not rare. Forced into survival jobs, educated migrants can also be affected by deskilling when their mobility grants end and they must work for a living with no possibility to transfer their academic credentials in their new environment. Traumatic experiences of lack of recognition, disrespect and inhumane treatment in the workplace may reinforce the vulnerability of incoming migrants. Additionally, the language barrier is a challenge for all WMWs, which educated migrants may nevertheless overcome with more ease than less educated ones. Daily prejudices and exclusion strategies from national-born workers are common whatever the education background.

The detrimental impact of deskilling and differential treatments affecting WMWs, as well as the missed opportunities for receiving countries, are not rare. Deskilling can often be attributed to racist and gendered practices of exclusion from the labour market and is detrimental to the migrants facing stressful integration conditions as well as the receiving nation that deprives itself from valuable skills ( Siar, 2013).

## WEMov’s recommendations

In keeping with a 2017 UN Women report, WEMov believes that policy-makers should be better informed on gender and migration, but also that women migrants should be involved in policy making, and their contributions to sending and receiving nations’ development acknowledged ( UN Women, 2017).

In light of the above study, WEMov recommends the following provisions and improvements:

### Highlighting women migrants’ skills and contributions to the EU

1) WEMov believes that it is necessary to change the macro-narrative from burden economic trope to value the diversity of women migrants’ backgrounds and skills. To impact the general narrative on WMWs and ease their integration, studies about migrants’ skills should be widely released;
2) Skills and labour should be degendered;
3) Care work and “lesser-skilled” jobs should be revalued by highlighting soft/non-educational skills ;
4) Defining skills in terms of receiving nations’ needs should be avoided, and migrants’ resources highlighted. Skills should be introduced in the interview process to avoid excluding the migrants deemed unnecessary, but rather start from the skills they have, not the receiving nation’s needs in order to promote diversity of skills and inclusionary practices;
5) Women migrants’ economic empowerment should be acknowledged as their increasing role as breadwinners and contribution to remittances, so global economy;
6) An intersectional approach to deskilling is necessary to understand and introduce adequate best practices, because other factors than education (gender, race, motherhood, country of origin) may impact women migrants’ employability;
7) Returnees’ skills should be recognised to facilitate their reintegration.

### Curbing deskilling

8) WEMov believes that it is important to make information available to women migrants on training and employment;
9) Access to decent work should be guaranteed for women migrants;
10) Reskilling should be encouraged with specific trainings (language and vocational) accessible to women migrants;
11) EU protection tools against women migrants’ exploitation and fair recruitment policies should be developed, and recruitment agencies monitored to avoid exploitation: deskilling and exploitation often being linked;
12) Gender segmentation at work confining women migrants to reproductive unskilled jobs should be curbed;
13) Efficient qualification and diplomas equivalence procedures for EU and non-EU in-migrants should be set up;
14) Labour marker integration practices should be harmonized across EU Member states to limit the overrepresentation of WMWs’ deskilling in Southern Member States.
15) The language and motherhood penalties should be addressed with language courses and childcare provisions. Childcare should be provided for to ease women migrants’ employment;
16) WMWs’ access to the same career advancement opportunities as national-born women workers should be ensured;
17) Curbing the gender pay gap would benefit all women workers, but labour protection and equal pay should be guaranteed to WMWs, who should be given access to training and skills development ( UN Women, 2017).

## Conclusion

Deskilling translates into a devaluation of previously acquired qualifications, experience and expertise in the receiving nation when taking up lower-status jobs. It is proven that highly skilled women migrate more than men, yet non-EU born women face higher risks of unemployment, lower paid employment, exploitation and abuse ( EIGE, 2020). So-called “trailer” migrants, i.e. accompanying spouses, often face deskilling because they are assigned to a dependent status with traditional gendered family responsibilities and thus see their professional qualifications devalued. Skills in the migration context is defined in terms of who is a desirable migrant and who is less desirable in the receiving country. A key element in deskilling is the devaluation of qualifications obtained in the Global South such as Africa or Asia. Hence, WMWs may be discriminated against because of their country of origin as well as linguistic barriers. They thus face discriminations that reflect global economic development, world economic dynamics and deskilling being tied ( IOM, 2015).

The main issue that WEMov has identified with WMWs’ deskilling is the ensuing de-emancipation of women migrants. WMWs’ exclusion from quality decent and protected work conditions generates social tensions and entrenches gender labour divisions. WMWs’ integration can also be constrained by the cost of their migration, which prevents them from accessing training and reskilling and rather confines them to subsistence employment. Hence, deskilled WMWs are at a higher risk of exploitation, physical and sexual violence in the workplace ( UN Women, 2024).

However, migration can lead to deskilling as well as reskilling, and commentators have noticed that migration can be both empowering for women ( EIGE, 2020) (upskilling) and disempowering (deskilling). Women are often greater contributors to remittances, either in financial or social terms, social remittances concerning the transmission of cultural and social values, beyond the money that women transfer back home, which underlines women migrants’ constructive contribution to their departing and destination societies ( UN Women, 2017). Therefore, receiving nations benefit from the arrival of educated migrants, and sending nations may also benefit from returnees’ skills acquired in migration. As such, the migration process can be identified as producer of skills. Yet, although education can allow women to achieve economic empowerment, upskilling and reskilling initiatives have generally not ended gender discriminations in the labour market ( UN Women, 2024).

## Ethical approval and consent

Ethical approval and consent were not required.

## Data Availability

No data are associated with this article.
