# Supplementary material for: Women migrant workers’ (WMWS) deskilling
Source: Open Res Eur. 2025 Jun 18;4:186. Originally published 2024 Aug 21. [Version 2] doi: 10.12688/openreseurope.18205.2 (PMC12203307; doi:10.12688/openreseurope.18205.2)
Supplement: Peer review history

# Reviewer report: Reviewer response for version 2

**DOI:** 10.21956/openreseurope.22037.r55689

**Published:** 2025-08-09

**Authors:** Sara Verderber

**Affiliations:** 1 University of Ljubljana, Ljubljana, Slovenia

I have read the revised version of the article, which flows clearly and coherently. The theoretical part has been strengthened and clarified, the data sector has been structured, and the clarity of the reading has also improved. The authors have taken recommendations and given comments into account. I have no reservations about approving the article. 

 The topic is significant and underexplored, so I greatly welcome the scientific input of the authors of this article once again.

Where applicable, are recommendations and next steps explained clearly for others to follow? (Please consider whether others in the research community would be able to implement guidelines or recommendations and/or constructively engage in the debate)

Yes

Does the article adequately reference differing views and opinions?

Partly

Are all factual statements correct, and are statements and arguments made adequately supported by citations?

Partly

Is the rationale for the Open Letter provided in sufficient detail? (Please consider whether existing challenges in the field are outlined clearly and whether the purpose of the letter is explained)

Yes

Is the Open Letter written in accessible language? (Please consider whether all subject-specific terms, concepts and abbreviations are explained)

Yes

Reviewer Expertise:

women's migration, professional identity, highly-skilled migration, integration, and mental health.

I confirm that I have read this submission and believe that I have an appropriate level of expertise to confirm that it is of an acceptable scientific standard.

# Reviewer report: Reviewer response for version 2

**DOI:** 10.21956/openreseurope.22037.r55687

**Published:** 2025-07-23

**Authors:** Parvati Raghuram

**Affiliations:** 1 The Open University, Milton Keynes, England, UK

I recommend that the article be indexed without any further revisions.

Where applicable, are recommendations and next steps explained clearly for others to follow? (Please consider whether others in the research community would be able to implement guidelines or recommendations and/or constructively engage in the debate)

Yes

Does the article adequately reference differing views and opinions?

Partly

Are all factual statements correct, and are statements and arguments made adequately supported by citations?

Partly

Is the rationale for the Open Letter provided in sufficient detail? (Please consider whether existing challenges in the field are outlined clearly and whether the purpose of the letter is explained)

Yes

Is the Open Letter written in accessible language? (Please consider whether all subject-specific terms, concepts and abbreviations are explained)

Yes

Reviewer Expertise:

NA

I confirm that I have read this submission and believe that I have an appropriate level of expertise to confirm that it is of an acceptable scientific standard.

# Reviewer report: Reviewer response for version 2

**DOI:** 10.21956/openreseurope.22037.r55688

**Published:** 2025-06-25

**Authors:** Maria Bastida

**Affiliations:** 1 University of Santiago de Compostela, Santiago de Compostela, Spain

I have read the revised version of the article and reviewed the authors' response to my comments. I believe that the new version has been considerably improved, with clearer conceptual distinctions, better contextualisation and a more coherent structure. The specific points I raised, particularly with regard to regional comparisons and employment indicators, have been addressed satisfactorily.

 For my part, I consider the revised article ready for indexed.

Where applicable, are recommendations and next steps explained clearly for others to follow? (Please consider whether others in the research community would be able to implement guidelines or recommendations and/or constructively engage in the debate)

Yes

Does the article adequately reference differing views and opinions?

No

Are all factual statements correct, and are statements and arguments made adequately supported by citations?

Partly

Is the rationale for the Open Letter provided in sufficient detail? (Please consider whether existing challenges in the field are outlined clearly and whether the purpose of the letter is explained)

Yes

Is the Open Letter written in accessible language? (Please consider whether all subject-specific terms, concepts and abbreviations are explained)

Yes

Reviewer Expertise:

NA

I confirm that I have read this submission and believe that I have an appropriate level of expertise to confirm that it is of an acceptable scientific standard.

# Reviewer report: Reviewer response for version 1

**DOI:** 10.21956/openreseurope.19675.r44396

**Published:** 2024-10-17

**Authors:** Sara Verderber

**Affiliations:** 1 University of Ljubljana, Ljubljana, Slovenia

The article "Women migrant workers' (WMWS) deskilling" focuses on the important but often neglected topic of women's migration. It is an important academic contribution because research on women's migrant experience is still rare. Secondly, existing research usually focuses on women in the context of family reunification (so-called 'trailing migrants') or as care workers and low-skilled labour, while high-skilled women migrants rarely receive academic attention in migration studies.

 This article represents a significant development in the migration academic field by exploring migration structures and realities in the post-migration phase through the prism of gender and qualification (education). It highlights the importance of a research focus on women migrant workers facing deskilling. It explores how deskilling can directly affect migrant women and what indirect implications this concept has on the host country's economy and society development. As both a personal experience and a political and economic concept, deskilling encompasses a broad spectrum of migrant labour processes and is a common challenge for highly skilled migrant women, with necessary macro-level implications, such as the potential loss of economic potential and skills/knowledge for both: the societies of arrival and for the migrant workers themselves. The authors successfully place migrant experience at the centre of the debate, which offers an understanding of the impact of deskilling on the socio-political and economic structures of arrival and departure communities. However, the article needs additional clarifications: a better explanation of used concepts is required, the academic references are often missing, and basic theoretical support is necessary to contextualise the content adequately.

 If we look more closely, there are insufficient breakdowns of concepts such as "glass ceiling," "gender pay gap," and "global south" (although even people from the global north are not immune to deskilling too). There is a lack of relevant academic references to back up claims such as "affect women migrant workers in greater numbers than men," "deskilling is generally a result of administrative barriers," and "linguistic barriers." Although the article includes several data and explanations on the concept of deskilling and the current legal frameworks in European migrant labour legislation and regulation, these elements are not linked well. The shortcomings are also reflected in the lack of theoretical links and explanations that would clarify how the concepts of gender and racial discrimination and language proficiency affect deskilling. The data presented, although scattered throughout the text, is not well placed in the context of deskilling, racism, integration, and gender (including pay) discrimination, which reduces the coherence of the analysis.

 To improve the article, it would be helpful to:

- Increase the coherence and systematicity of the text.
- Include academic references for the claims made.
- Interpret and relate the data more precisely to the deskilling topic.
- Add theoretical background or background to help situate the data in the discussion.

While the paper addresses a topical and important issue, it is clear that there is a binding need for additional clarity and a structured approach to linking the concepts mentioned to deskilling and the data set.

Where applicable, are recommendations and next steps explained clearly for others to follow? (Please consider whether others in the research community would be able to implement guidelines or recommendations and/or constructively engage in the debate)

Yes

Does the article adequately reference differing views and opinions?

Partly

Are all factual statements correct, and are statements and arguments made adequately supported by citations?

Partly

Is the rationale for the Open Letter provided in sufficient detail? (Please consider whether existing challenges in the field are outlined clearly and whether the purpose of the letter is explained)

Yes

Is the Open Letter written in accessible language? (Please consider whether all subject-specific terms, concepts and abbreviations are explained)

Yes

Reviewer Expertise:

women's migration, professional identity, highly-skilled migration, integration, and mental health.

I confirm that I have read this submission and believe that I have an appropriate level of expertise to confirm that it is of an acceptable scientific standard, however I have significant reservations, as outlined above.

## References

[1] : The professional identity of highly educated migrant women: a multinational study of migration processes from a gender and educational perspective. *European Social Work Research* .2024;2(1) : 10.1332/27551768Y2024D000000010 69-87 10.1332/27551768Y2024D000000010

[2] : Duševno zdravje visoko izobraženih migrantk. *Socialno Delo* . 10.51741/sd.2023.62.1.29-42 10.51741/sd.2023.62.1.29-42

# Response

**Published:** 2025-05-02

**Authors:** Marie-José Ruiz

**Affiliations:** Anglophone Studies, Universite de Picardie Jules Verne, Amiens, Hauts-de-France, France

We thank the reviewer for their engaged and generous evaluation of our policy brief, and for recognising its contribution to the underrepresented field of highly skilled women migrant workers (WMWs). We are especially appreciative of the reviewer’s acknowledgement of our effort to centre migrant women’s experiences and to explore the broader socio-political and economic implications of deskilling.

**1. Conceptual Clarifications** We fully agree that key terms such as **“glass ceiling,” “gender pay gap,” “Global South,”** and “linguistic barriers” require further clarification to ensure accessibility for a broad academic and policy audience. Concepts like **gendered labour segmentation**, **intersectional discrimination**, and **migration policy restrictions** interrelate and shape WMWs’ experiences of deskilling. For example, “glass ceiling” includes gendered and racialised implications in the context of career progression, while “Global South” is qualified in relation to structural inequities and postcolonial patterns in migration systems.

**2. Improved Data Interpretation and Linkages** Statistical references are **contextually tied to analytical claims** about deskilling, labour market integration, or legal frameworks. Connections between **data on overqualification, gender pay gaps, and sectoral employment** and the broader issues of skill recognition, discriminatory hiring practices, and structural barriers are clear.

**3. Improved Coherence and Structure** We thank the reviewer for pointing out the need for greater **systematicity**. The document structure to follow a **clear progression** from definition and context → evidence and challenges → case studies → policy recommendations. **Final Remarks** We are grateful for the reviewer’s thoughtful engagement and their recognition of the paper’s importance in expanding the academic conversation on gender and skilled migration.

**Marie Ruiz & Stellamarina Donato**

# Reviewer report: Reviewer response for version 1

**DOI:** 10.21956/openreseurope.19675.r44395

**Published:** 2024-10-04

**Authors:** Parvati Raghuram

**Affiliations:** 1 The Open University, Milton Keynes, England, UK

This is a concise and important article on deskilling of women migrant workers. It focuses particularly on the European case. It clearly sets out the instruments available to address the problems and has a useful list of recommendations. It is therefore a  paper that will benefit the readership of this journal and I recommend that it be approved for indexing after the authors address the suggestions listed below.

 I would, however, suggest a few changes to the paper. I found the second paragraph of the first section 'WEMov's approach' really useful for context. I could not see why this was not given in the beginning of the paper along with what WEMov is. The underpinning research of WEMov was not clearly set out in the beginning. What are the countries in which this network is based and what is its aim?

 The section WEMov's case studies did not give me information on the specific case studies - whether they be sectors which they studied, or the countries on which they focus. This would have helped the reader to understand the project. From the paragraph it appears that the work may be focused on psychological impact, in which case the recommendations should align with this. It currently does not focus on the psychological recommendations.

 These two sections also don't make clear how the project goes beyond the existing literature and debates. What is the new value added through WEMov?

 I would like to have seen some examples of countries or sectors where the deskilling is not as much as in others, why that might have been and what could have been learnt from it. There is a vast amount of data on this already. For instance, Canada and Australia vary in the extent to which ethnicity rather than country of qualification influence deskilling. There are also differences in the nature of deskilling based on the sector. In male dominated sectors such as IT and engineering, it is the limited participation of national women in these sectors that leads to a lot of difficulties for migrant women as the recruiters struggle to accept that women could be in these sectors, there is a lot of machoism and male privilege amongst workers and there are difficulties for the companies recruiting them to accommodate women. You can see more details of all this in my publication which I have provided references for below. (Ref - 1 and Ref - 2)

 It would also have been useful to have a little more clarity on how far these are national problems or labour market sector problems. The relative role of immigraiton regulations compared to labour market regulations  affects the nature of deskilling. For instance, some people may be able to enter the sector in which they are trained but not at the same level, while others have no access to that sector. There is also a role for employment agencies, which often assume that migrant women are looking for work in particular sectors. You may find some of these differences, as they pertain to Indian migrant women in the EU in this publication Raghuram, Parvati (2022 [Ref-1]). *Indian Women's Migration to the EU.* International Labour Office, Switzerland.

 Moving forward it would be good to think about how we go beyond some tropes around deskilling and how we look at skilled migration through a wider lens. For instance, our study found that some women wanted to use the opportunity offered by migration to not work in the sector they trained in because they wanted to do other things, which they found more fulfilling. While deskilling is a pressing concern, perhaps a footnote to say that not all women who do not join the labour market are unhappy with this because, for some, the higher salaries that their partners earn in the EU, might provide a relief from the double burden that they faced as skilled women in countries with lower salaries.

 On a minor note, there are a few places where the paper could do with an edit.

 Here is one:

 'Quantitative data confirm that in 2019, 1/5 highly educated WMWs experienced deskilling: 40.7% for migrant women compared to 21.1% for national women in 2020.'. I think there might be some errors in this statement.

 Overall, an important topic and I hope the authors will consider these comments as ways in which to take this work forward in the future and to get even more readership for their paper. I wish the authors all the very best in this endeavour

Where applicable, are recommendations and next steps explained clearly for others to follow? (Please consider whether others in the research community would be able to implement guidelines or recommendations and/or constructively engage in the debate)

Yes

Does the article adequately reference differing views and opinions?

Partly

Are all factual statements correct, and are statements and arguments made adequately supported by citations?

Partly

Is the rationale for the Open Letter provided in sufficient detail? (Please consider whether existing challenges in the field are outlined clearly and whether the purpose of the letter is explained)

Yes

Is the Open Letter written in accessible language? (Please consider whether all subject-specific terms, concepts and abbreviations are explained)

Yes

Reviewer Expertise:

Gendered migration, skilled migration, IT sector, nursing and medicine, care and domestic work, EU.

I confirm that I have read this submission and believe that I have an appropriate level of expertise to confirm that it is of an acceptable scientific standard, however I have significant reservations, as outlined above.

## References

[1] : Indian Women's Migration to the EU. *The Open University* .2022; Reference source

[2] : Indian migrant women in the European Union labour market: beyond stereotypes and current tropes. *The Open University* .2024;13:25-33 Reference source

# Response

**Published:** 2025-05-02

**Authors:** Marie-José Ruiz

**Affiliations:** Anglophone Studies, Universite de Picardie Jules Verne, Amiens, Hauts-de-France, France

We are grateful for the reviewer’s thoughtful engagement with our policy brief and for recognising the significance of the topic, the relevance of our recommendations, and the value of the contribution to ongoing policy debates. We appreciate the specific and constructive suggestions. **1. Clarifying WEMov’s Research Foundation and Scope** We fully agree that the *WEMov* network’s background and research scope needs to be integrated at the start of the brief. The **geographical scope** of WEMov spans over 30 European and neighbouring countries and the **aim** of the network is to highlight migrant women’s contributions and improving gender-sensitive migration policies. **2. WEMov Case Studies – Clarification and Focus** The **locations** of the three featured cases are Ireland, Sweden, and Poland. The **sectors** are hospitality, recruitment, education, tourism, and factory work. The approach was **qualitative, based on interviews**, with attention to both labour market trajectories and **psychological impacts**, particularly burnout, devaluation, and frustration resulting from deskilling. The psychological dimensions were not the initial focus of the recommendations, **3. WEMov’s Contribution Beyond Existing Literature** WEMov adds value by documenting the **historical, geographic, and gendered dimensions** of deskilling through a multi-country lens. It contributes **original interviews and qualitative insights** to complement the large body of quantitative data already available. Our policy brief identifies **intersections between deskilling and EU-level instruments**, not often addressed in feminist migration literature. This positions WEMov explicitly within, and beyond, current academic and policy debates. **4. Positive Deviance: Where Deskilling Is Less Prevalent** There are examples of regions with **lower levels of deskilling**. The section on Portugal explores **why it performs better** than other Southern European countries, noting **less restrictive equivalency systems** and a stronger presence of migrant-led networks. Comparative references to **Canada and Australia** note how differences in credential recognition and sectoral integration models influence outcomes. **5. Sector-Specific Gender Bias and Deskilling** D **eskilling in male-dominated sectors like IT and engineering** is reinforced by gendered stereotypes among recruiters ; lack of inclusive hiring practices ; and cultural “machoism” in the workplace that can alienate migrant women. This nuance complements the earlier discussion on gender segmentation in care and service work and acknowledges different patterns of deskilling depending on the sector. **6. Distinguishing Labour Market vs. Immigration Policy Effects** S **ectoral constraints (e.g., professional licensing)** can limit access to skilled roles, while i **mmigration policies**, especially tied visas or conditional work permits, can create precarity and further reinforce deskilling. **7. Beyond the Deskilling Trope: Migrant Women’s Agency** Not all WMWs view a career change or withdrawal from the labour market as negative. In some cases, migration offers a reprieve from the double burden of work and care in home countries. These choices reflect agency and **diverse aspirations**, which should be acknowledged in future work. We agree this is an important area for further research and are grateful for the reminder not to homogenise experiences of deskilling. **8. Editing and Clarification of Statistical Statements** We thank the reviewer for flagging the unclear sentences. We also completed a minor language edit throughout to enhance readability. We again thank the reviewer for the constructive feedback and collegial tone. **Marie Ruiz & Stellamarina Donato**

# Reviewer report: Reviewer response for version 1

**DOI:** 10.21956/openreseurope.19675.r43543

**Published:** 2024-09-09

**Authors:** Maria Bastida

**Affiliations:** 1 University of Santiago de Compostela, Santiago de Compostela, Spain

The paper examines the key role of women migrant workers in the development of both sending and receiving countries. Despite their important contributions, women migrant workers face labour de-skilling, which involves under-utilisation of their skills and lack of recognition of their qualifications. Factors such as lack of equivalence of qualifications, language barriers and ethnic and gender stereotypes exacerbate this problem. Work overload has a negative impact on the social mobility and well-being of these women, while at the same time preventing receiving countries from making full use of their skills.

 The report outlines international and European policies and frameworks that seek to protect the rights of women migrant workers, while recognising the need to redouble efforts to effectively implement these policies. I found the report interesting because it addresses a current and pressing problem that disproportionately affects women.

 In my opinion, the document provides a comprehensive overview of the de-skilling of women migrant workers, covering the political, social and economic factors that contribute to this situation, with a particular focus on international and EU regulations and policies. The inclusion of data and statistics, such as deskilling figures and gender pay gaps, provides a clear basis for analysis. These figures help to contextualise the extent of the problem in the different regions of Europe, providing a basis for the report's conclusions and recommendations. In addition, the value of interpersonal skills acquired through the migration process is recognised, providing an important perspective on the non-traditional contributions of women workers to the human capital of receiving countries. Finally, the report includes practical recommendations that address structural issues affecting migrant women's labour market integration.

 However, the report lacks specific academic references that corroborate the current state of research. The inclusion of previous studies on the subject would help to validate the arguments presented and strengthen the theoretical basis. Some data, such as the lower incidence of the problem in Portugal, are not sufficiently explained. It would be useful to explore further the reasons for this difference compared to other southern European countries. In addition, it should be clarified whether the employment rates of immigrant women refer to the total number of employees in a sector or to the total number of immigrant women employed.

 Although mention is made of sectors in which migrant women are over-represented, such as health and care, the report does not explore internal differences within these sectors. For example, working as a doctor, nurse or health assistant involves different roles, and these nuances could enrich the analysis of labour segmentation. The report also fails to adequately address the similarities between the employment sectors of home-based workers and native women. Women, in general, are highly represented in the service sector. It would be relevant to reflect on whether WMWs face additional factors that worsen their employment situation or whether they share the same challenges as non-migrant women.

 The paper could also benefit from an analysis of the skills available in receiving countries. For example, if native-born women have low skill levels, WMWs could theoretically have more job opportunities. This factor could influence the degree of de-skilling experienced by migrant women workers.

 The section on the ‘skills and resources’ of women domestic workers offers no more than a subjective view of their resourcefulness, without clearly defining what is meant by this concept or how it is measured. A more rigorous analysis of the skills these women bring and how they can be harnessed in host countries would be beneficial.

 The case study section does not differentiate the situation of female migrants from that of male migrants. Everything stated in the final sections could also be applied to male migrants. The report could benefit from more specificity in identifying the unique challenges faced by migrant women in the workplace, rather than generalising problems shared with men.

**Suggestions for improvement**

- Expand the academic references to strengthen the state of the research and provide a stronger theoretical basis.
- Provide more detailed explanations of some data, such as the situation in Portugal, and clarify employment rates to avoid confusion.
- Include a more detailed analysis of employment sectors and internal segmentation, especially in areas such as health and care.
- Reflect on the similarities between immigrant and non-immigrant women in terms of employment and analyse whether there are additional factors that worsen the situation of immigrant women.
- Analyse the skills available in receiving countries to assess whether the skill levels of native women offer advantages or disadvantages to WMWs in labour market integration.
- Revise the section on ‘skills and resources’ with a more concrete and data-driven analysis.
- Differentiate the situation of female migrants from that of male migrants in the case studies to highlight the specific challenges faced by women.

Finally, the recommendations presented in the report may appear somewhat vague and generic in some respects. While they address important issues, they lack concrete details on how to implement the proposals or measure their effectiveness.

 I hope this report is useful and thank you for your confidence in my judgement.

Where applicable, are recommendations and next steps explained clearly for others to follow? (Please consider whether others in the research community would be able to implement guidelines or recommendations and/or constructively engage in the debate)

Yes

Does the article adequately reference differing views and opinions?

No

Are all factual statements correct, and are statements and arguments made adequately supported by citations?

Partly

Is the rationale for the Open Letter provided in sufficient detail? (Please consider whether existing challenges in the field are outlined clearly and whether the purpose of the letter is explained)

Yes

Is the Open Letter written in accessible language? (Please consider whether all subject-specific terms, concepts and abbreviations are explained)

Yes

Reviewer Expertise:

In general terms, everything to do with women's professional careers (business, entrepreneurship, social economy, expatriation).

I confirm that I have read this submission and believe that I have an appropriate level of expertise to confirm that it is of an acceptable scientific standard, however I have significant reservations, as outlined above.

# Response

**Published:** 2025-05-02

**Authors:** Marie-José Ruiz

**Affiliations:** Anglophone Studies, Universite de Picardie Jules Verne, Amiens, Hauts-de-France, France

We sincerely thank the reviewer for their detailed and thoughtful evaluation of our policy brief. We are pleased that the report is recognised as timely, relevant, and practically oriented, particularly in its emphasis on the systemic nature of deskilling and the underutilisation of women migrant workers’ (WMWs) skills.

**1. Academic References and Theoretical Framework** We fully agree about the inclusion of **scholarly references**, our data points and conclusions are informed by both academic research and fieldwork from WEMov COST Action.

**2. Clarifying Regional Discrepancies ** We thank the reviewer for pointing out the need to better explain the **lower deskilling rate in Portugal** compared to other Southern European countries.

**3. Clarification of Employment Rate Metrics** Specifying w **hat the employment rates refer to**—namely, whether they pertain to WMWs as a share of a sector or of the total employed migrant women—avoids ambiguity.

**4. Internal Segmentation in Health and Care Sectors** We appreciate the reviewer’s suggestion to differentiate between roles within **health and care sectors**, and we note the **institutional barriers to recognition** that often prevent WMWs from practicing in line with their qualifications, which enhances the understanding of **horizontal and vertical segmentation** in feminised labour sectors.

**5. Comparison with Native Women’s Employment Patterns** We agree that it is important to reflect on the **commonalities and differences between migrant and non-migrant women**. The **compounded challenges faced by WMWs**—language barriers, legal status, and racism—exacerbate these shared disadvantages.

**6. Skills Available in Receiving Countries** N **ative women's skill levels** are impacted by labour market conditions in receiving countries (e.g., skill shortages) which could mitigate deskilling for WMWs. The paradox of high-skilled migrant women is **systematically channelled into low-skill sectors**, regardless of local skill supply.

**7. ‘Skills and Resources’ Section** We appreciate the feedback on this section and the difference between “ **soft skills**” and “resourcefulness”.

**8. Differentiating Gendered Experiences in Case Studies** We **explicitly highlight how the challenges faced by WMWs differ from those of male migrants**, with emphasis on gendered labour expectations; vulnerability to exploitation in feminised jobs; discriminatory migration sponsorship tied to family roles. The case studies provide **gender-specific insight**, not just general migrant experiences. We are grateful for your thoughtful assessment and we hope to offer foundation for advancing fairer policies and practices toward women migrant workers in Europe. **Marie Ruiz & Stellamarina Donato**
